# Olfactory inputs regulate *Drosophila melanogaster* oogenesis

**DOI:** 10.1242/jeb.247234

**Published:** 2024-12-11

**Authors:** Madhumala K. Sadanandappa, Giovanni Bosco

**Affiliations:** Department of Molecular and Systems Biology, Geisel School of Medicine at Dartmouth, Hanover, NH 03755, USA

**Keywords:** Germ stem cells, Olfaction, Olfactory receptors, Fecundity, Proliferation, Sensory signaling

## Abstract

*Drosophila* female germline development and maintenance require both local stem cell niche signaling and systemic regulation. Here, we show the indispensable function of the *Drosophila melanogaster* olfactory circuit in normal oogenesis and fecundity. Lack of olfactory inputs during development causes a reduction in germline stem cells. Although germline stem cells proliferate normally, the germline cysts undergo caspase-mediated apoptosis, leading to decreased follicle production and egg-laying in flies with defective olfaction. Strikingly, activation of olfactory circuits is sufficient to boost egg production, demonstrating that chemosensory-activated brain-derived inputs promote gamete development. Given the energy demands of oogenesis and its direct consequence on fitness, we propose that olfactory-stimulated systemic regulation evolved tightly with downstream diet-responsive pathways to control germline physiology in response to nutritional status. Additionally, these findings raise the possibility that sensory-mediated stem cell maintenance is a generalizable mechanism spanning a myriad of neuronal circuits, systems and species.

## Teaser

**Highlighted Article:** Although olfactory systems are essential for chemosensing environmental and social cues, sensory perception and its deprivation can also affect oogenesis and fecundity in *Drosophila melanogaster*.

## INTRODUCTION

Olfaction is one of the most ancient, evolutionarily critical physiological systems, and it plays a pivotal role in the survival and fitness of most animals. Besides identifying sources of food, mating partners and oviposition sites, and discerning predator threats, olfactory-mediated responses can also have long-term impacts on species, including transgenerational effects (Deshe et al., 2023; Dias and Ressler, 2014; Pinto, 2011). Given the critical role of olfaction in the continuous environmental assessment of animals, we asked whether olfactory stimulation regulates the development and maintenance of non-neuronal tissues such as gametes. Precedence for this idea can be found in *Drosophila* larvae, where normal development of the immune system relies on olfactory signaling to hematopoietic progenitors in the lymph gland, demonstrating chemosensory inputs in hematopoiesis and innate immune response (Madhwal et al., 2020; Shim et al., 2013).

**Fig. 1.**
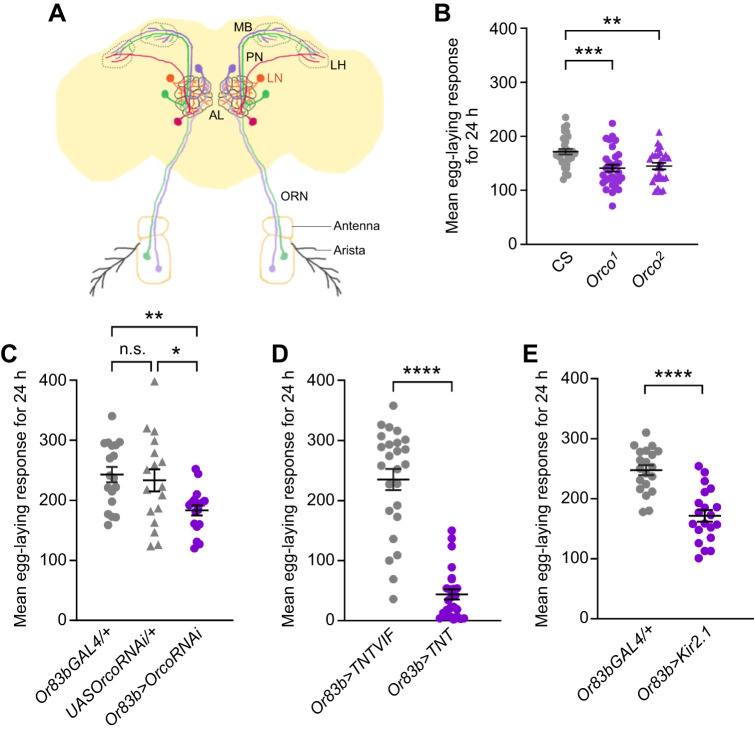
**Flies with defective olfaction show reduced fecundity.** (A) Schematic of *Drosophila melanogaster* olfactory system. ORN, olfactory receptor neuron; PN, projection neuron; LN, local interneuron; MB, mushroom body; LH, lateral horn; AL, antennal lobe. (B–E) Dot plots showing 24 h mean egg-laying responses of (B) *Orco* mutants (*Orco^1^* and *Orco^2^*), and flies expressing (C) RNAi against *Orco* receptor (*Or83b>OrcoRNAi*), (D) either an inactive (*Or83b>TNTVIF*) or active form of tetanus toxin light chain (*Or83b>TNT*) and (E) *Kir_2.1_* channel (*Or83b>Kir_2.1_*) in ORNs. Data are means±s.e.m. from at least two to three independent experiments (*P<0.05, **P<0.01, ***P<0.001, ****P<0.0001, n.s. *P*>0.05).

Owing to its well-studied, relatively simple olfactory circuit and the availability of genetic toolkits that allow *in vivo* analysis, we investigated germline development in *Drosophila melanogaster* with defective olfaction (Su et al., 2009; Vosshall and Stocker, 2007). Briefly, in adult flies, the antennae and maxillary palp are two specialized peripheral sensory appendages that bear olfactory receptor neurons (ORNs) (Fig. 1A). Activation of odor-specific olfactory receptors (ORs) expressed in these ORNs stimulates the second-order projection neurons in the antennal lobe, which in turn relay information to higher brain centers – the mushroom body and lateral horn (Stocker et al., 1990). Within the antennal lobe, the local interneurons of diverse morphology, function and neurotransmitter identity fine-tune the olfactory information, thereby modulating the physiology and behavioral outputs resulting from the sensation of specific odorants (Chou et al., 2010; Seki et al., 2010).

**Fig. 2.**
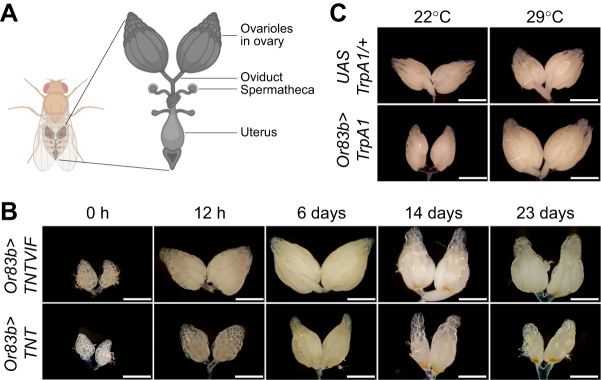
**Ovary development requires olfactory inputs.** (A) Schematic of *D. melanogaster* female reproductive tract. (B) Micrographs of whole-mount ovaries from *Or83b>TNTVIF* and *Or83b>TNT* visualized at indicated time points after eclosion. Note that mated females were used for whole-mount ovary analysis, except for newly emerged females at 0 h. (C) Representative ovary images of *UASTrpA1/+* and *Or83b>TrpA1* maintained at 22°C and 29°C. Scale bars: 100 μm.

**Fig. 3.**
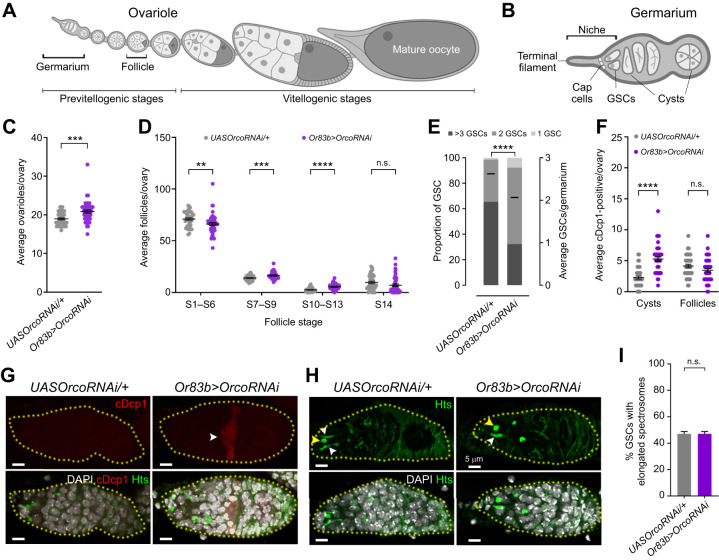
**Olfactory cues regulate oogenesis.** Diagrams of a *D. melanogaster* (A) ovariole with an anterior germarium followed by developing follicles and (B) germarium. Number of (C) ovarioles and (D) different stages of developing follicles in *UASOrcoRNAi*/+ (*n*=38) and *Or83b>OrcoRNAi* ovaries (*n*=48). (E) Frequencies of germaria containing one, two or more germline stem cells (GSCs) in *UASOrcoRNAi*/+ (*n*=281) and *Or83b>OrcoRNAi* flies (*n*=347). The black line corresponds to the average number of GSCs per germarium. (F) Average number of cDcp1-positive germline cysts and follicles in *UASOrcoRNAi*/+ (*n*=38) and *Or83b>OrcoRNAi* ovaries (*n*=48). (G) A representative germarium (outlined in yellow) labeled with DAPI (white) nuclei; Hts (green) spectrosomes and fusomes; and cDcp1 (red) apoptotic cells. The white arrowhead indicates cDcp1-positive germline cyst in *Or83b>OrcoRNAi* germaria. (H) A confocal image of germarium (outlined in yellow) labeled with DAPI (white) and Hts (green). The yellow and white arrowheads indicate GSCs with round and elongated spectrosomes, respectively. Scale bars (G,H): 5 μm. (I) Percentage of GSCs with elongated spectrosomes in *UASOrcoRNAi*/+ (*n*=796) and *Or83b>OrcoRNAi* germarium (*n*=813). *UASOrcoRNAi/+* and *Or83b>OrcoRNAi* are presented in grey and purple, respectively. Data are means±s.e.m. from three independent experiments (**P<0.01, ***P<0.001, ****P<0.0001, n.s. *P*>0.05).

The *D. melanogaster* germline, with its well-described biology, offers an excellent system for studying gametogenesis, including how germline stem cells (GSCs), along with their lineages, receive information from and respond to various environmental and physiological inputs (Drummond-Barbosa, 2019; Spradling, 1993). Each fly ovary is subdivided into a functional unit called an ovariole (Figs 2A and 3A). The anterior tip of each ovariole houses a stem cell niche, including two to three GSCs, terminal filament cells, cap cells and escort cells (Fig. 3B) (Fuller and Spradling, 2007). When GSCs divide asymmetrically, they self-renew and produce cystoblasts (CBs), daughter cells destined for differentiation. The CB undergoes four rounds of mitotic divisions with incomplete cytokinesis to generate an interconnected 16-cell germline cyst, of which one cell will eventually give rise to an oocyte, and the remaining cells become supporting nurse cells. A newly formed 16-cell germline cyst subsequently enveloped by somatic follicle cells buds off from the germarium posteriorly forming an egg chamber, which develops through 14 stages of oogenesis, including the onset of yolk update at stage 8 (vitellogenesis) and forming a matured oocyte, to be fertilized and deposited (Drummond-Barbosa, 2019; Fuller and Spradling, 2007).

Although previous studies reported reduced fecundity in olfactory mutants and germline expression of OR genes (David et al., 2023; Dubey et al., 2016; Liu et al., 2023; Spehr et al., 2003; Yan et al., 2017), the precise role of olfactory cues in female germline development and maintenance remain unclear. By employing a neurogenetic and immunohistochemistry analysis, we uncover the olfactory control of GSC maintenance, follicle development and fertility in *D. melanogaster*. By establishing a link between olfactory perception and oogenesis, we discuss the broader implications of olfactory-stimulated systemic regulation of physiology, particularly the advantages of regulating stem cell populations.

## MATERIALS AND METHODS

### Flies and fly husbandry

*Drosophila melanogaster* Meigen 1830 stocks were cultured on a standard cornmeal medium (Sadanandappa et al., 2023) at 25°C temperature under 12 h:12 h light:dark cycle-controlled conditions, and Canton S (CS) flies were used as wild-type controls unless otherwise stated. *Or83bGAL4* (III) (Larsson et al., 2004; Weaver et al., 2020) stock was provided by Mani Ramaswami (Trinity College, Dublin, Ireland). *Orco* null mutants (III) *Orco^1^* (no. 23129) and *Orco^2^* (no. 23130) (Larsson et al., 2004), *UASTNTVIF* (II) (no. 28840), *UASTNT* (II) (no. 28838) (Martin et al., 2002; Sweeney et al., 1995), *UASKir_2.1_* (II) (no. 6596) (Hardie et al., 2001) and *UASTrpA1* (II) (no. 26263) (Hamada et al., 2008) stocks were from Bloomington *Drosophila* Stock Center, and *UASOrcoRNAi* (II) (KK100825) was from the Vienna *Drosophila* Resource Center.

### Egg-laying assay

Following CO_2_ anesthetization, age-synchronized (6 days post-eclosion) flies, five females and two males, were placed in a vial containing fresh fly medium. After 24 h of egg-laying in an incubator with controlled conditions (25°C and 12 h:12 h light:dark cycle), all flies were removed before manually documenting the number of eggs in each vial using a ZEISS Stemi 2000 stereomicroscope (Sadanandappa et al., 2023).

For *Or83b>TrpA1* experiments, the crosses were grown at 22°C. Two days post-eclosion, the experimental flies were shifted to 29°C for 4 days, whereas the age-matched controls were maintained at 22°C. Genotype control flies (*UASTrpA1/+*) were handled similarly.

### Tissue preparation and immunohistochemistry

Ovaries were dissected from mated females and immunolabeled using a previously described protocol (Sadanandappa et al., 2021). In this study, we used mouse anti-Hts 1B1 (1:50, Developmental Studies Hybridoma Bank), rabbit cleaved anti-Dcp1 (1:200, Cell Signaling Technology, no. 9578) and Alexa Fluor 488- or 568-conjugated secondary antibodies (1:400, Molecular Probes). Immunolabeled ovaries were mounted in Vectashield (H-1000, Vector Laboratories, Newark, CA, USA), and confocal images were acquired with a Nikon A1R confocal microscope. For image processing, we used Nikon's NIS-Elements and Adobe Photoshop software.

We used previously described methods to analyze the ovaries. (1) To identify and quantify different follicle stages, we visualized the DAPI-stained ovaries using a Nikon Eclipse E800 microscope (20× magnification) (Hudson and Cooley, 2014; Spradling, 1993). (2) Based on their proximity to the niche and spectrosome labeling by anti-Hts antibody, GSCs were identified and quantified. (3) Dividing GSCs were identified by elongated spectrosomes, whereas non-proliferative GSCs have round spectrosomes (Fig. 3H) (de Cuevas and Spradling, 1998). (4) Further, we used the apoptotic marker cDcp1 to quantify cell death in the germline cysts and developing follicles (McCall, 2004; Sarkissian et al., 2014).

### Statistical analysis

GraphPad Prism (version 9.4.1) was used to analyze data and plot the graphs. Mean values were derived from two to three independent experiments and the error bars correspond to ±s.e.m. Statistical significance was determined as follows: *P<0.05, **P<0.01, ***P<0.001, ****P<0.0001 and n.s. for non-significance (*P*>0.05). The Results section includes mean±s.e.m., sample number (*n*), details of the statistical tests along with corresponding *P-*values for all quantifications.

## RESULTS AND DISCUSSION

*Drosophila melanogaster* ORNs express a conserved odorant co-receptor called *Orco*, which forms a heterodimeric complex with conventional ORs to detect specific odors and activate the olfactory circuit (Fig. 1A) (Su et al., 2009; Vosshall and Stocker, 2007). Homozygous *Orco* null mutants, *Orco^1^* and *Orco^2^*, generated by a gene-targeted technique replacing the portions of the *Orco* coding region with the *white* gene, produce viable and fertile adult progeny (Larsson et al., 2004). However, *Orco* mutant larvae and adult flies are defective in odorant-mediated behavioral and electrophysiological responses, including olfactory learning and memory (McGuire et al., 2005). We assessed 24 h of egg-laying in age-matched Canton S (CS), a wild-type *D. melanogaster* strain, and *Orco* mutants maintained at controlled conditions. Both *Orco^1^* (141.60±6.60, *n=*30, *P*<0.001) and *Orco^2^* mutants (144.92±5.93, *n*=26, *P*=0.004) showed a significant reduction in egg-laying compared with wild-type controls (171.60±5.15, *n*=30, one-way ANOVA with Bonferroni's correction) (Fig. 1B).

To account for any potential effects of the *Orco* mutant's genetic background (Larsson et al., 2004), we employed the GAL4-UAS binary system to perturb the *D. melanogaster* olfactory circuit (Brand and Perrimon, 1993). First, we performed RNAi-mediated *Orco* knockdown using *Or83bGAL4*, an ORN-specific driver (Larsson et al., 2004; Weaver et al., 2020). *Or83b>OrcoRNAi* (183.16±8.48, *n*=18) phenocopied *Orco* mutant phenotype for egg-laying (*Or83bGAL4/+*, 242.78±12.70, *n*=18, *P*=0.0086 and *UASOrcoRNAi/+*, 233.18±18.31, *n*=17, *P*=0.037, one-way ANOVA with Bonferroni's correction) (Fig. 1C). Next, we disrupted the synaptic function of ORNs by expressing the tetanus toxin transgene (*Or83b>TNT*), which significantly reduces spontaneous release and abolishes evoked neurotransmitter release by enzymatically cleaving synaptobrevin (Martin et al., 2002; Sweeney et al., 1995). Compared with flies expressing an inactive form of tetanus toxin in ORNs (*Or83b>TNTVIF*, 234.88±17.53, *n*=25), females expressing an active form of tetanus toxin (*Or83b>TNT*, 43.88±8.57, *n*=25, *P*<0.0001, unpaired *t*-test) mimic the *Orco* mutant phenotype for egg-laying (Fig. 1D). Finally, we silenced ORNs by expressing *Kir_2.1_* (*Or83b>Kir_2.1_*), a human inward rectifier K^+^ channel that inhibits the generation of an action potential by hyperpolarizing neuronal membranes (Baines et al., 2001). *Or83b>Kir_2.1_* egg-laying data further confirmed reduced fecundity in flies with olfactory defects (*Or83bGAL4/+*, 247.50±8.24, *n*=20 and *Or83b>Kir_2.1_*, 171.55±9.81, *n*=20, *P*<0.001, unpaired *t*-test) (Fig. 1E). Thus, ORN activity is important for promoting normal oogenesis rather than the mere absence of OR function.

We systematically examined the germline development to determine how the olfactory circuit affects oogenesis. *Drosophila melanogaster* females have two ovaries, each comprising 16–20 ovarioles containing GSCs at the anterior end, followed by developing follicles (Figs 2A and 3A) (Spradling, 1993). A newly eclosed female ovary is mainly composed of previtellogenic follicles and undergoes reproductive maturation 2–3 days post-eclosion (Bodenstein, 1947; Spradling, 1993). As expected, we observed predominately previtellogenic follicles in the whole-mount ovary preparations of newly eclosed *Or83b>TNTVIF* and *Or83b>TNT* flies (Fig. 2B). However, 12 h post-eclosion, *Or83b>TNTVIF* ovaries developed typically comprising vitellogenic follicles, whereas *Or83b>TNT* ovaries remained relatively small with early follicle stages (Fig. 2B).

The above observation prompted us to ask whether olfactory perturbation delays follicle development leading to reduced egg production (Fig. 1). Therefore, we examined ovaries on different post-eclosion days (Fig. 2B), including the reproductive peak (6–12 days post-eclosion) and senescence period of *D. melanogaster* females (Miller et al., 2014; Spradling, 1993). Six-day-old, mated *Or83b>TNTVIF* female ovaries were composed of more matured vitellogenic follicles than *Or83b>TNT* flies (Fig. 2B; Fig. S3). *Orco* mutants, *Or83b>OrcoRNAi* and *Or83b>Kir_2.1_* ovaries also exhibited similar germline physiology (Figs S1–S3). The whole-mount ovary analysis of aged females (post-eclosion days 14 and 23) revealed diminished ovary size with fewer follicles in *Or83b>TNT* females, thus supporting a role for olfaction in promoting normal oogenesis (Fig. 2B).

To further support this conclusion, we directly depolarized sensory neurons by transgenic expression of *TrpA1*, a heat-activated transient receptor potential family ion channel, in ORNs (*Or83b>TrpA1*) (Das et al., 2011; Pulver et al., 2009; Rosenzweig et al., 2005). Temperature-induced activation of cation permeable channel in *Or83b>TrpA1* flies was sufficient to increase ovary size and number of developing follicles at high temperature (29°C) compared with control flies maintained at 22°C and *UASTrpA1/+* females at 29°C (Fig. 2C; Fig. S3), thus confirming the olfactory regulation of *D. melanogaster* oogenesis.

Each *D. melanogaster* ovariole contains two or three GSCs at the most anterior end, which divide to generate progenitors that eventually develop into eggs (Fig. 3A,B). Under optimum environmental conditions, ovarioles can continuously produce eggs. The number of ovarioles per ovary, established during larval ovarian development, is crucial for determining fecundity (Büning, 1994; Sarikaya et al., 2012). Surprisingly, *Orco* knockdown flies with reduced fecundity have an increased number of ovarioles (20.85±0.39, *n*=48) compared with controls (*UASOrcoRNAi*/+, 18.94±0.27, *n*=38, *P*<0.001, unpaired *t*-test with Welch's correction) (Fig. 3C; Fig. S1C). However, the quantification of different follicle stages in *Or83b>OrcoRNAi* showed a significant reduction in follicle stages S1 to S6 (*UASOrcoRNAi/+*, 71±1.17, *n*=38 and *Or83b>OrcoRNAi*, 66.08±1.37, *n*=48, *P*=0.010, unpaired *t*-test with Welch's correction) (Fig. 3D).

Further analysis of the germarium in terms of GSC number and cell death substantiates the reduced follicles and fecundity observed in *Or83b>OrcoRNAi* females (Fig. 3E–I; Fig. S1D,E). First, we identified the GSCs and their daughter cells, CBs, based on their proximity to the niche and adducin staining using anti-Hu-li tai shao (Hts) (Fig. 3H). At the germarium tip, GSCs contact niche cap cells with a prominent adducin focus on the anterior part, labeling the subcellular structure called spectrosomes. Second, older CBs located outside the niche show a branched focus of adducin in the posterior part, known as a fusome (de Cuevas and Spradling, 1998). *Or83b>OrcoRNAi* ovaries showed a reduction in the average number of GSCs per germanium (*UASOrcoRNAi*/+, 2.75±0.04, *n*=281 and *Or83b>OrcoRNAi*, 2.19±0.03, *n*=347, *P*<0.0001, unpaired *t*-test) (Fig. 3E).

Next, we quantified the cell death by assaying the ovary expression of cleaved Dcp1, an effector of *Drosophila* caspase (McCall, 2004; Tatevik et al., 2014). We documented increased apoptosis of germline cysts in *Or83b>OrcoRNAi* germarium (*UASOrcoRNAi*/+, 2.29±0.25, *n*=38 and *Or83b>OrcoRNAi*, 5.27±0.32, *n*=48, *P*<0.0001, unpaired *t*-test with Welch's correction) compared with developing follicles (*UASOrcoRNAi*/+, 4.13±0.28, *n*=38 and *Or83b>OrcoRNAi*, 3.44±0.27, *n*=48, *P*=0.172, unpaired *t*-test with Welch's correction) (Fig. 3F,G; Figs S1D,E and S3). Finally, we examined GSC proliferation by assessing a distinct spectrosome morphology – round or elongated – indicating the cell cycle of stem cells (de Cuevas and Spradling, 1998) (Fig. 3G). The number of GSCs with elongated spectrosomes in the control and *Orco* knockdown germarium are indistinguishable (*UASOrcoRNAi*/+, 47.11±1.77, *n*=796 and *Or83b>OrcoRNAi*, 47.11±1.75, *n*=813, *P*=0.999, unpaired *t*-test) (Fig. 3H,I), demonstrating olfactory regulation of GSC maintenance and follicle development, not GSC proliferation.

Together, our results suggest that activation of brain-derived olfactory circuits regulates egg production through hormonal and/or neuronal mechanisms rather than through direct function of ORs per se. Female *Drosophila* maintained on a poor diet show a germline physiology similar to that of olfactory mutants (Drummond-Barbosa and Spradling, 2001; Hsu and Drummond-Barbosa, 2009; Lin and Hsu, 2020). These dietary responses are regulated by highly conserved metabolic pathways such as *Drosophila* insulin-like peptides (DILPs), adipocyte factors, Target of Rapamycin (TOR) and AMP-dependent kinase (AMPK), as well as extensive interorgan communication (Drummond-Barbosa, 2019). These similarities suggest that olfactory signaling may engage the diet-dependent pathways in the downstream circuit to regulate oogenesis, which warrants further screening and characterization of OR-specific circuit mechanisms. Because oogenesis demands high energy and resources, these mechanisms have likely evolved tightly to adjust reproductive responses based on olfactory cues that convey environmental information, including nutrients and threats. The regulation of oogenesis in *Caenorhabditis elegans* by chemosensory neurons in response to food-derived cues via insulin signaling provides additional evidence in line with our observations (Mishra et al., 2023).

### Conclusions

The crucial role of olfactory inputs in the normal development of female gametes in *D. melanogaster* raises the possibility that active chemosensation could be a general mechanism for maintaining stem cells and developing tissues. For example, the olfactory-mediated GABA release from larval neurosecretory cells into the hemolymph binds to the GABA_B_ receptors in the lymph gland of *D. melanogaster*. This binding elevates cytosolic calcium levels, which is necessary and sufficient for maintaining hematopoietic progenitors and triggering the innate immune response (Madhwal et al., 2020; Shim et al., 2013). Of fundamental importance, these studies suggest that other sensory perception systems, such as visual, gustatory, tactile and auditory systems, might similarly control development of non-neuronal tissues and adult stem cell populations.

## Supplementary Material

10.1242/jexbio.247234_sup1 Supplementary information
