# Supplementary material for: Olfactory inputs regulate Drosophila melanogaster oogenesis
Source: J Exp Biol. 2024 Dec 11;227(24):jeb247234. doi: 10.1242/jeb.247234 (PMC11655025; doi:10.1242/jeb.247234)
Supplement: Supplementary information [file jexbio-227-247234-s1.pdf]

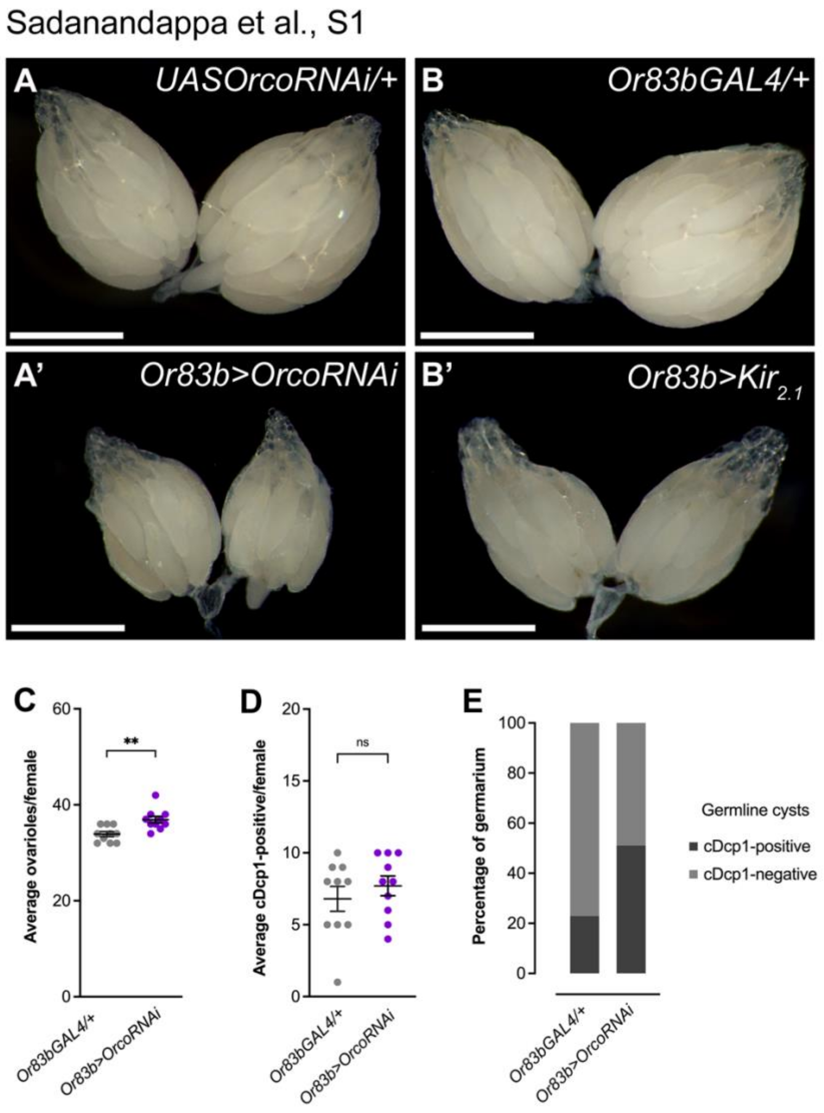

Fig. S1. Representative whole mount ovary images of (A) *Or83b>OrcoRNAi* and (B) *Or83b>Kir*$_{2.1}$ along with their respective controls. Scale bar 500 µm. Average number of (C) ovarioles and (D) cDcp1-positive follicles in *Or83bGAL4/+* (n = 10) and *Or83b>OrcoRNAi* females (n = 10). (E) Percentage of germaria containing cDcp1-positive (dark grey) or cDcp1-negative (light grey) germline cyst in *Or83bGAL4/+* (n = 306) and *Or83b>OrcoRNAi* flies (n = 329). *Or83bGAL4/+* and *Or83b>OrcoRNAi* are presented in grey and purple, respectively, with mean ± SEM and $^{**}p < 0.01$ and ns for non-significance determined by *t*-test.

Sadanandappa et al., S2

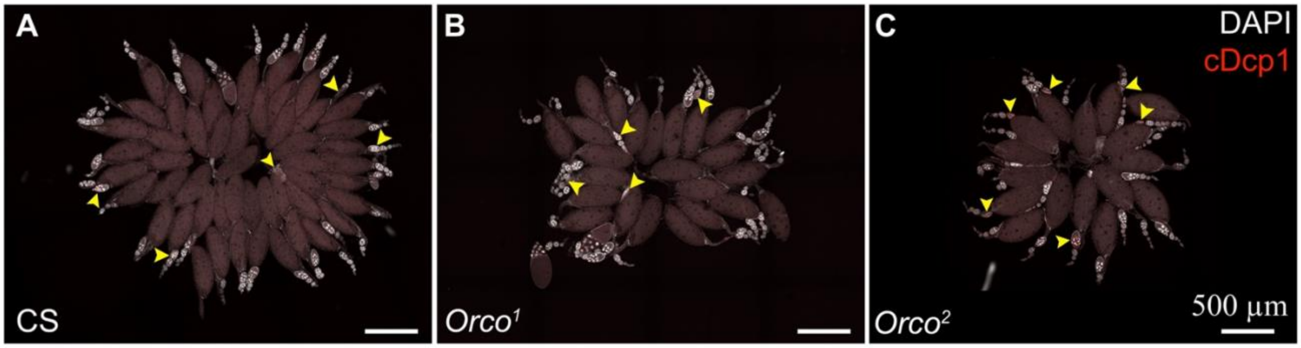

Fig. S2. Representative images of ovaries labeled with cDcp1 (red) and DAPI (white) in (A) CS, (B) *Orco*^{1} and (C) *Orco*^{2} flies. Scale bar 500 µm. The yellow arrowheads indicate cDcp1-positive apoptotic follicles.

Journal of Experimental Biology • Supplementary information

Sadanandappa et al., S3

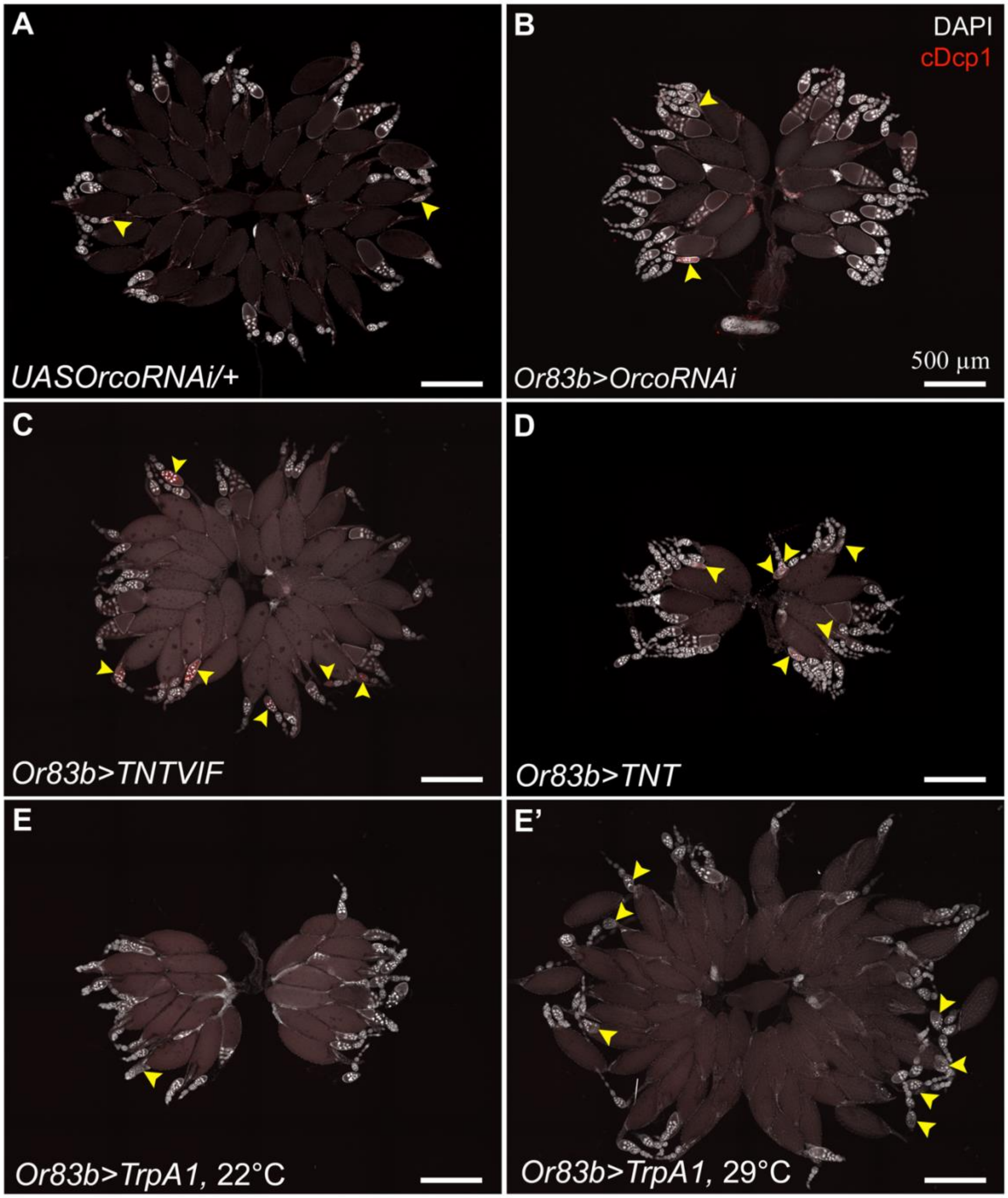

Fig. S3. Representative images of ovaries labeled with cDcp1 (red) and DAPI (white) in (A) *UASOrcoRNAi/+*, (B) *Or83b>OrcoRNAi*, (C) *Or83b>TNTVIF*, (D) *Or83b>TNT*, and (E and E') *Or83b>TrpA1* flies maintained at 22ºC and 29ºC. Scale bar 500 µm. The yellow arrowheads indicate cDcp1-positive apoptotic follicles.
